# Comparative cardiovascular outcomes in type 2 diabetes patients taking dapagliflozin versus empagliflozin: a nationwide population-based cohort study

**DOI:** 10.1186/s12933-023-01911-7

**Published:** 2023-07-26

**Authors:** Jaehyun Lim, You-Jung Choi, Bong Sung Kim, Tae-Min Rhee, Hyun-Jung Lee, Kyung-Do Han, Jun-Bean Park, Jin Oh Na, Yong-Jin Kim, Heesun Lee, Hyung-Kwan Kim

**Affiliations:** 1 Department of Internal Medicine, Seoul National University Hospital, Seoul, Republic of Korea; 2 Department of Internal Medicine, Seoul National University College of Medicine, Seoul, Republic of Korea; 3 Division of Cardiology, Department of Internal Medicine, Korea University College of Medicine, Seoul, Republic of Korea; 4 Department of Statistics and Actuarial Science, Soongsil University, Seoul, Republic of Korea; 5 Division of Cardiology, Department of Internal Medicine, Seoul National University Hospital Healthcare System Gangnam Center, 152, Teheran-ro, Gangnam-gu,, Seoul, Republic of Korea; 6 Diagnostic Test Unit Section of Cardiovascular Imaging, Division of Cardiology, Cardiovascular Center, Department of Internal Medicine, Seoul National University Hospital,, Seoul National University College of Medicine, 101 Daehak-ro, Jongno-gu, 03080 Seoul, Korea

**Keywords:** Sodium-glucose co-transporter-2 inhibitors, Dapagliflozin, Empagliflozin, Cardiovascular risk, Heart failure, Cardiovascular death

## Abstract

**Background:**

Sodium-glucose co-transporter-2 inhibitors displayed cardiovascular benefits in type 2 diabetes mellitus in previous studies; however, there were some heterogeneities regarding respective cardiovascular outcomes within the class. Furthermore, their efficacies in Asians, females, and those with low cardiovascular risks were under-represented. Thus, we compared the cardiovascular outcomes between new users of dapagliflozin and empagliflozin in a broad range of patients with type 2 diabetes mellitus using a nationwide population-based real-world cohort from Korea.

**Methods:**

Korean National Health Insurance registry data between May 2016 and December 2018 were extracted, and an active-comparator new-user design was applied. The primary outcome was a composite of heart failure (HF)-related events (i.e., hospitalization for HF and HF-related death), myocardial infarction, ischemic stroke, and cardiovascular death. The secondary outcomes were individual components of the primary outcome.

**Results:**

A total of 366,031 new users of dapagliflozin or empagliflozin were identified. After 1:1 nearest-neighbor propensity score matching, 72,752 individuals (mean age approximately 56 years, 42% women) from each group were included in the final analysis, with a follow-up of 150,000 ~ person-years. Approximately 40% of the patients included in the study had type 2 diabetes mellitus as their sole cardiovascular risk factor, with no other risk factors. The risk of the primary outcome was not different significantly between dapagliflozin and empagliflozin users (hazard ratio [HR] 0.93, 95% confidence interval [CI] 0.855–1.006). The risks of secondary outcomes were also similar, with the exception of the risks of HF-related events (HR 0.84, 95% CI 0.714–0.989) and cardiovascular death (HR 0.76, 95% CI 0.618–0.921), which were significantly lower in the dapagliflozin users.

**Conclusions:**

This large-scale nationwide population-based real-world cohort study revealed no significant difference in composite cardiovascular outcomes between new users of dapagliflozin and empagliflozin. However, dapagliflozin might be associated with lower risks of hospitalization or death due to HF and cardiovascular death than empagliflozin in Asian patients with type 2 diabetes mellitus.

**Supplementary Information:**

The online version contains supplementary material available at 10.1186/s12933-023-01911-7.

## Introduction

Type 2 diabetes mellitus (T2DM) is a common metabolic disorder affecting over 462 million people worldwide, for whom one of the most crucial goals is to improve cardiovascular outcomes [1]. The sodium-glucose co-transporter-2 (SGLT2) inhibitors, a relatively new oral glucose-lowering drug (GLD) class, have been demonstrated to significantly improve cardiovascular outcomes compared with placebo or other GLDs in patients with T2DM [2–6]. This class of medication continues to be at the forefront of extensive research, positioning them as the most widely investigated drugs in the contemporary treatment era [7–10]. Given that the difference in hemoglobin A1c (HbA1c) improvement between SGLT2 inhibitor users and controls was modest in previous large, randomized, placebo-controlled, cardiovascular outcome trials (CVOTs), the cardiovascular benefits provided by SGLT2 inhibitors are thought to be attributed to both systemic actions, such as glycosuria and natriuresis, and direct cardiac effects, including the attenuation of cardiac inflammation, oxidative stress, and mitochondrial dysfunction, rather than its direct glucose-lowering effects [2–4, 11].

Dapagliflozin and empagliflozin are representative SGLT2 inhibitors that are widely prescribed worldwide. However, clinical trials with these two medications showed inconsistencies, especially regarding cardiovascular death [2–4, 12–15]. Whether drug-specific differences exist within the class is not fully understood. Meta-analyses also concluded that directly comparing different SGLT2 inhibitors through previous CVOTs would be limited due to varying study populations enrolled in each CVOT [16, 17]. Hence, they emphasized the need for further head-to-head comparison studies. In addition, Asians, females, and patients with low cardiovascular risks other than T2DM have been under-represented in previous studies. Furthermore, observational studies to date have mostly focused on the glycemic or metabolic efficacy of SGLT2 inhibitors, such as improvement in body weight, blood pressure, fasting plasma glucose, HbA1c, or serum lipid profiles [18–24]. Since the cardiovascular benefits of SGLT2 inhibitors stem mainly from their pleiotropic rather than direct glucose-lowering effects and intensive glucose control may not result in cardiovascular benefits, a study that focuses more on the role of SGLT2 inhibitors as a modifier of cardiovascular outcomes is required [25–28]. Therefore, we compared the risks of cardiovascular outcomes between dapagliflozin and empagliflozin in patients with T2DM using a well-established nationwide cohort in Korea.

## Methods

### Study design and database

This nationwide population-based cohort study was conducted using the database from the Korean National Health Insurance Service (NHIS) and the NHIS-Health Screening Program. In Korea, the NHIS is a social health insurance service and medical aid covering medical costs for the entire population [29]. The Korean NHIS database contains sociodemographic information and health insurance data for outpatient visits or hospitalization [29]. Individuals’ medical information is recorded based on the International Classification of Diseases, Tenth Revision (ICD-10) codes. The NHIS-Health Screening Program database includes data on health check-ups such as physical examinations, blood pressure, body mass index (BMI), regular blood tests, and self-reported questionnaires on lifestyle, such as smoking, alcohol consumption, and physical activity. The database, including the information above, was then merged with death records provided by Statistics Korea. Consequently, our data included the full spectrum of relevant outcomes of interest. The characteristics and validity of the NHIS database have been described elsewhere [29, 30].

The institutional review board of Seoul National University Hospital exempted the study protocol from review (IRB No: 2003-006-1105) because the NHIS database is publicly available to facilitate research with anonymity and de-identified information. The study complied with the tenets of the Declaration of Helsinki, and we followed the Strengthening the Reporting of Observational Studies in Epidemiology (STROBE) reporting guideline.

### Study Population

**Fig. 1:**
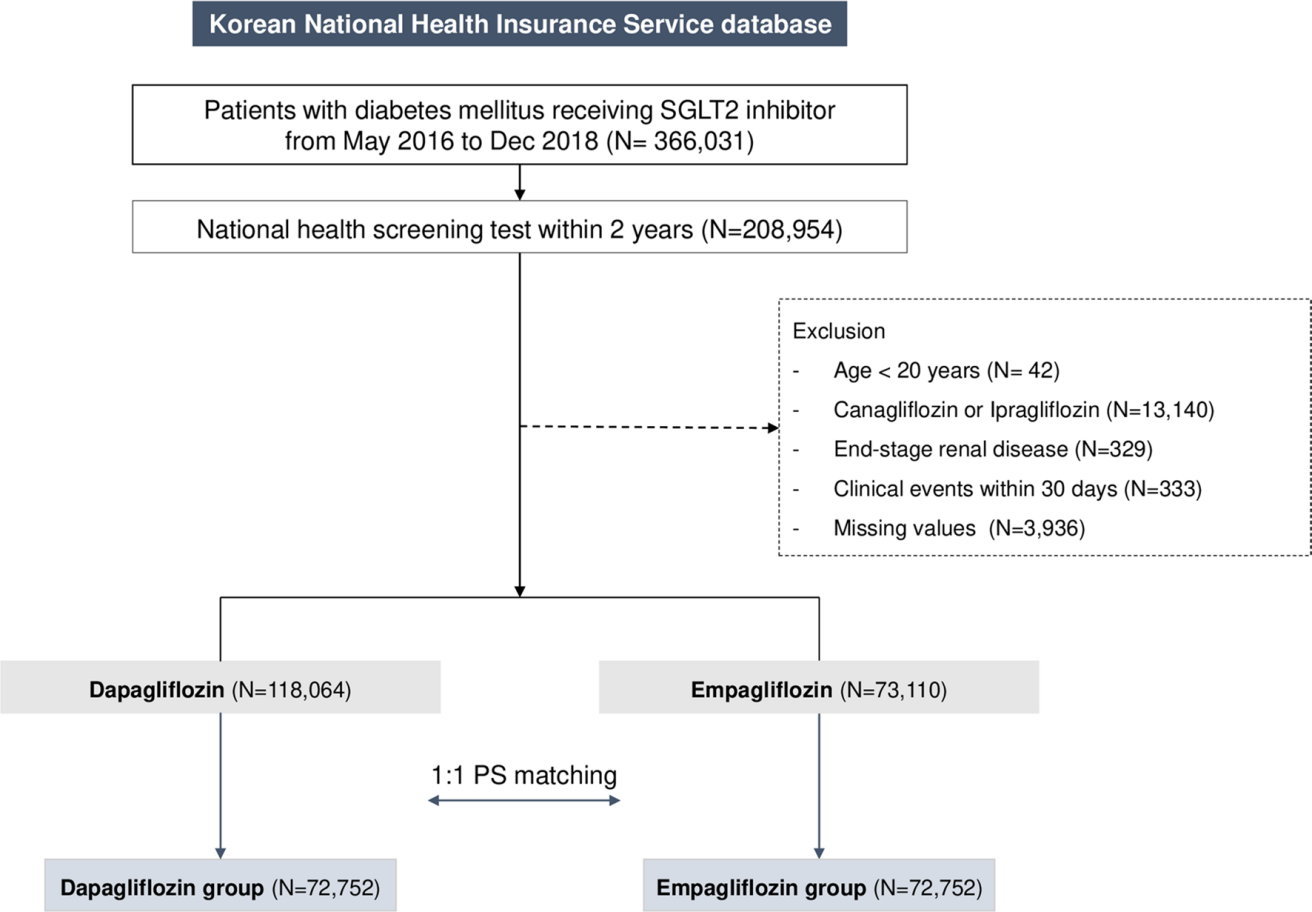
Flow chart presenting the selection process of the study population. Patients with type 2 diabetes newly using SLGT2 inhibitors were selected from the Korean National Health Insurance Service database. Dapagliflozin users were 1:1 matched to empagliflozin users using propensity score matching. * SGLT2* sodium-glucose co-transporter 2

From the NHIS database, we identified 366,031 patients diagnosed with T2DM who started taking SGLT2 inhibitors between May 2016 and December 2018. Of note, dapagliflozin and empagliflozin were approved for insurance coverage in January and May 2016, respectively, allowing for broader utilization. T2DM was defined based on the diagnostic codes for T2DM (ICD-10 codes E11–E14): the one recorded during hospitalization or at least two outpatient clinic recordings ± the prescription of insulin, glucagon-like peptide-1 agonist, or at least one GLD [30]. GLDs included metformin, sulfonylurea, meglitinides, thiazolidinediones, dipeptidyl peptidase-4 inhibitors, and alpha-glucosidase inhibitors. We applied an active-comparator, new-user design. Data on health check-ups were obtained from the NHIS-Health Screening Program database taken within two years before and closest to the date of SGLT2 inhibitor initiation. We excluded patients aged < 20 years, those taking SGLT2 inhibitors other than dapagliflozin or empagliflozin, and those diagnosed with end-stage renal disease. We also excluded patients who developed clinical events within 28 days after the index date, defined as the first date of SGLT2 inhibitor prescription. We excluded these patients because < 4 weeks of exposure to SGLT2 inhibitors was deemed unlikely to have caused the observed cardiovascular events. Finally, those with missing variables were also excluded (Fig. 1).

### Study outcomes

The primary outcome was a composite of heart failure-related events (hospitalization or death from heart failure), ischemic stroke, myocardial infarction (MI), and cardiovascular death. We included heart failure-related events as an additional component to the traditional three-point major adverse cardiovascular events, comprising ischemic stroke, MI, and cardiovascular death. This decision was based on the anticipated robust benefit of SGLT2 inhibitors regarding heart failure-related events, regardless of prior history of heart failure or atherosclerotic cardiovascular diseases [17]. Conversely, the atherosclerotic benefits of SGLT2 inhibitors appeared to be confined to those with established cardiovascular disease [17]. Given that our study included a broad range of patients with T2DM, we expanded the primary outcome to encompass heart failure-related events in addition to the traditional three-point major adverse cardiovascular events. The secondary outcomes were the individual components of the primary outcome. The safety outcomes were newly diagnosed end-stage renal disease, diabetic ketoacidosis, hypoglycemia, and genitourinary tract infection. The definitions of the outcomes are detailed in Additional file 1: Table S1. T2DM and outcomes definitions have been validated previously [31, 32], with high positive predictive values of > 94% for the primary diagnostic codes of major clinical outcomes in the Korean NHIS database [30, 33]. Patients were followed from the index date to primary outcome occurrence, death, or the end of the study period (December 2019), whichever occurred first. Emigration (withdrawal from the insurance program) before the primary outcome was considered a censored observation.

### Covariates

We collected information regarding the covariates that could affect study outcomes, including demographics (age, sex, income level, and residence), traditional cardiovascular risk factors (hypertension, dyslipidemia, heart failure, MI, peripheral artery disease, atrial fibrillation, and ischemic stroke), and other comorbidities (chronic obstructive pulmonary disease, liver cirrhosis, and hyperthyroidism). We also collected laboratory test results, including the estimated glomerular filtration rate, urine protein level using a dipstick test, fasting plasma glucose, total serum cholesterol, and serum hemoglobin. The operational definitions of the covariates in this study were drawn from those used in previous peer-reviewed journals and are described in more detail in the Additional file 1: Table S1 [30].

### Statistical analysis

Propensity score (PS) matching was performed at 1:1 for new users of dapagliflozin and empagliflozin. The nearest neighbor was selected without replacement to address the potential differences in baseline characteristics between both groups. Specifically, a PS was estimated for all the participants using a logistic regression fit for individuals, adjusting for index year and the 48 clinically relevant covariables listed in Table 1. Each dapagliflozin user was then matched to one empagliflozin user with a caliper for the nearest-neighbor matching. To examine the matching effectiveness, we computed absolute standardized differences (ASDs). A value < 10% and closer to zero demonstrated that the variable was balanced between both groups.

**Table 1:** Baseline characteristics of the study population

Variables	Empagliflozin	Dapagliflozin	ASD
	N = 72,752	N = 72,752	
Age, years	56.1 ± 11.1	55.8 ± 11.0	0.027
Sex, male	42,300 (58.1)	42,403 (58.3)	0.003
Index year			
2016	12,664 (17.41)	12,034 (16.54)	0.023
2017	31,230 (42.93)	32,412 (44.55)	0.033
2018	28,858 (39.67)	28,306 (38.91)	0.016
Systolic blood pressure, mmHg	128.3 ± 14.8	128.3 ± 14.9	0.003
Diastolic blood pressure, mmHg	78.6 ± 10.1	78.7 ± 10.0	0.007
Body mass index, kg/m ^2^	26.9 ± 4.0	26.9 ± 4.0	0.014
Duration of diabetes, years	7.1 ± 5.5	7.0 ± 5.5	0.027
Low income	14,722 (20.2)	14,711 (20.2)	0.001
Urban residents	31,151 (42.8)	31,040 (42.7)	0.003
Smoking status			
Never smoker	39,035 (53.7)	38,965 (53.6)	0.002
Ex-smoker	16,097 (22.1)	16,077 (22.1)	0.001
Current smoker	17,620 (24.2)	17,710 (24.3)	0.003
Alcohol drinking status			
Never drinker	42,604 (58.6)	42,427 (58.3)	0.005
Mild drinker (< 30 g/day)	24,067 (33.1)	24,157 (33.2)	0.003
Heavy drinker (≥ 30 g/day)	6081 (8.4)	6168 (8.5)	0.004
Regular exercise	15,177 (20.9)	15,110 (20.8)	0.002
Comorbidities			
Hypertension	42,455 (58.4)	41,905 (57.6)	0.015
Dyslipidemia	53,277 (73.2)	52,890 (72.7)	0.012
Heart failure	3723 (5.1)	3539 (4.9)	0.012
Myocardial infarction	2051 (2.8)	1865 (2.6)	0.016
Peripheral artery disease	16,281 (22.4)	16,018 (22.0)	0.009
Ischemic stroke	2074 (2.9)	2003 (2.8)	0.006
Atrial fibrillation	1893 (2.6)	1739 (2.4)	0.014
COPD	7068 (9.7)	6917 (9.5)	0.007
Liver cirrhosis	608 (0.8)	622 (0.9)	0.001
Hyperthyroidism	2172 (3.0)	2111 (2.9)	0.005
Medications			
ARB/ACE inhibitor	38,370 (52.7)	37,886 (52.1)	0.005
Beta-blocker	7896 (10.9)	7522 (10.3)	0.017
Calcium channel blocker	23,434 (32.2)	23,240 (31.9)	0.006
Diuretics	10,092 (13.9)	9878 (13.6)	0.008
GLD before SGLT2 inhibitor use			
Metformin	68,099 (93.6)	68,210 (93.8)	0.007
Sulfonylurea	39,391 (54.1)	38,980 (53.6)	0.011
Meglitinides	357 (0.5)	329 (0.5)	0.006
Thiazolidinedione	10,861 (14.9)	10,682 (14.7)	0.007
DPP4 inhibitor	45,776 (62.9)	45,476 (62.5)	0.009
α-glucosidase inhibitor	1544 (2.1)	1491 (2.1)	0.005
Insulin	10,615 (14.6)	10,668 (14.7)	0.002
GLP-1 agonist	626 (0.9)	608 (0.8)	0.002
Number of GLD ≥ 3	34,403 (47.3)	34,023 (46.8)	0.010
GLD combined with SGLT2 inhibitor			
Metformin	61,507 (84.5)	61,710 (84.8)	0.008
Sulfonylurea	28,394 (39.0)	28,182 (38.7)	0.006
Meglitinides	28 (0.04)	24 (0.03)	0.005
Thiazolidinedione	1221 (1.7)	1136 (1.6)	0.010
DPP4 inhibitor	5386 (7.4)	5528 (7.6)	0.008
α-glucosidase inhibitor	120 (0.2)	102 (0.1)	0.005
Insulin	5620 (7.7)	5846 (8.0)	0.012
GLP-1 agonist	51 (0.07)	60 (0.08)	0.004
Numbers of GLD ≥ 3	30,266 (41.6)	29,917 (41.1)	0.010
Estimated glomerular filtration rate	92.6 ± 48.2	92.9 ± 47.2	0.007
< 60 mL/min/1.73 m^2^	5358 (7.4)	4872 (6.7)	0.026
60–90 mL/min/1.73 m^2^	32,281 (44.4)	32,378 (44.5)	0.003
≥ 90 mL/min/1.73 m^2^	35,113 (48.3)	35,502 (48.8)	0.011
Urine protein by dipstick test			
Negative	62,278 (85.6)	62,300 (85.6)	0.001
Trace	3699 (5.1)	3824 (5.3)	0.008
Positive	6775 (9.3)	6628 (9.1)	0.007
Serum laboratory test			
Fasting plasma glucose, mg/dL	157.0 ± 55.2	157.5 ± 55.8	0.008
Total cholesterol, mg/dL	182.0 ± 46.0	183.0 ± 46.2	0.021
Hemoglobin, mg/dL	14.4 ± 1.6	14.4 ± 1.6	0.018

Data are presented as number (percentage) for categorical variables and mean ± standard deviation for continuous variables

*ACE* angiotensin-converting enzyme, *ASD* absolute standardized difference, *ARB* angiotensin II receptor blocker, *COPD* chronic obstructive pulmonary disease, *DPP4* dipeptidyl peptidase-4, *GLD* glucose-lowering drug, *GLP-1* glucagon-like peptide-1, *SGLT2 *sodium-glucose co-transporter-2

Baseline demographic data are presented as mean ± standard deviation or median (interquartile range) for continuous variables and as numbers (percentages) for categorical variables. Categorical variables were compared using the χ^2^ test or Fisher’s exact test. Continuous variables were compared using Student’s *t*-test or the Mann–Whitney U test. The incidence rate was the sum of all events divided by the total follow-up duration of 1000 person-years (PY). The incidence probability of the clinical outcomes was plotted using Kaplan–Meier curves with a statistical comparison using the log-rank test. Cox regression analysis assessed the association between SGLT2 inhibitors and the subsequent incidence of each outcome by estimating the hazard ratio (HR) with corresponding 95% confidence intervals (CIs).

We performed sensitivity analyses to confirm the robustness of our findings. First, we performed subgroup analyses including age (< 65 and ≥ 65 years), sex, insulin use (user and non-user), chronic kidney disease, duration of diabetes (< 7 and ≥ 7 years), and heart failure. We also performed a subgroup analysis by stratifying patients into three groups according to cardiovascular risks: (1) patients with established cardiovascular diseases (ischemic stroke, ischemic heart disease, MI, or peripheral artery disease), (2) patients with multiple cardiovascular risk factors (males ≥ 55 years or females ≥ 60 years with one or more traditional risk factors, including hypertension, dyslipidemia, or tobacco use), and (3) patients with low cardiovascular risks. We adjusted for the covariates used in the PS while conducting sensitivity analyses. Second, we analyzed individuals who continued to use the SGLT2 inhibitors without missing treatment days (as-treated approach) by censoring patients who skipped their medications for > 28 days. Third, we compared the non-cardiovascular mortality.

SAS software (version 9.4; SAS Institute Inc., Cary, NC, USA) was used for all statistical analyses. A *P*-value of < 0.05 was considered significant.

## Results

### Baseline characteristics

In total, 118,064 new users of dapagliflozin and 73,110 new users of empagliflozin were included. Before PS matching, dapagliflozin users were slightly younger, comprised a smaller proportion of beta-blocker users, had a higher total cholesterol level, and had an approximately 3 month shorter duration of diabetes compared to those in empagliflozin users (Additional file 1: Table S2). After PS matching with index year and the relevant covariates presented in Table 1, 72752 individuals (mean age 55.8 ± 11.0 years, 58.3% males for dapagliflozin; mean age 56.1 ± 11.1 years, 58.1% males for empagliflozin) were enrolled in each group for the final analysis. All baseline characteristics were well-balanced after PS matching (all ASDs < 5%, Table 1).

### Primary outcome

**Fig. 2:**
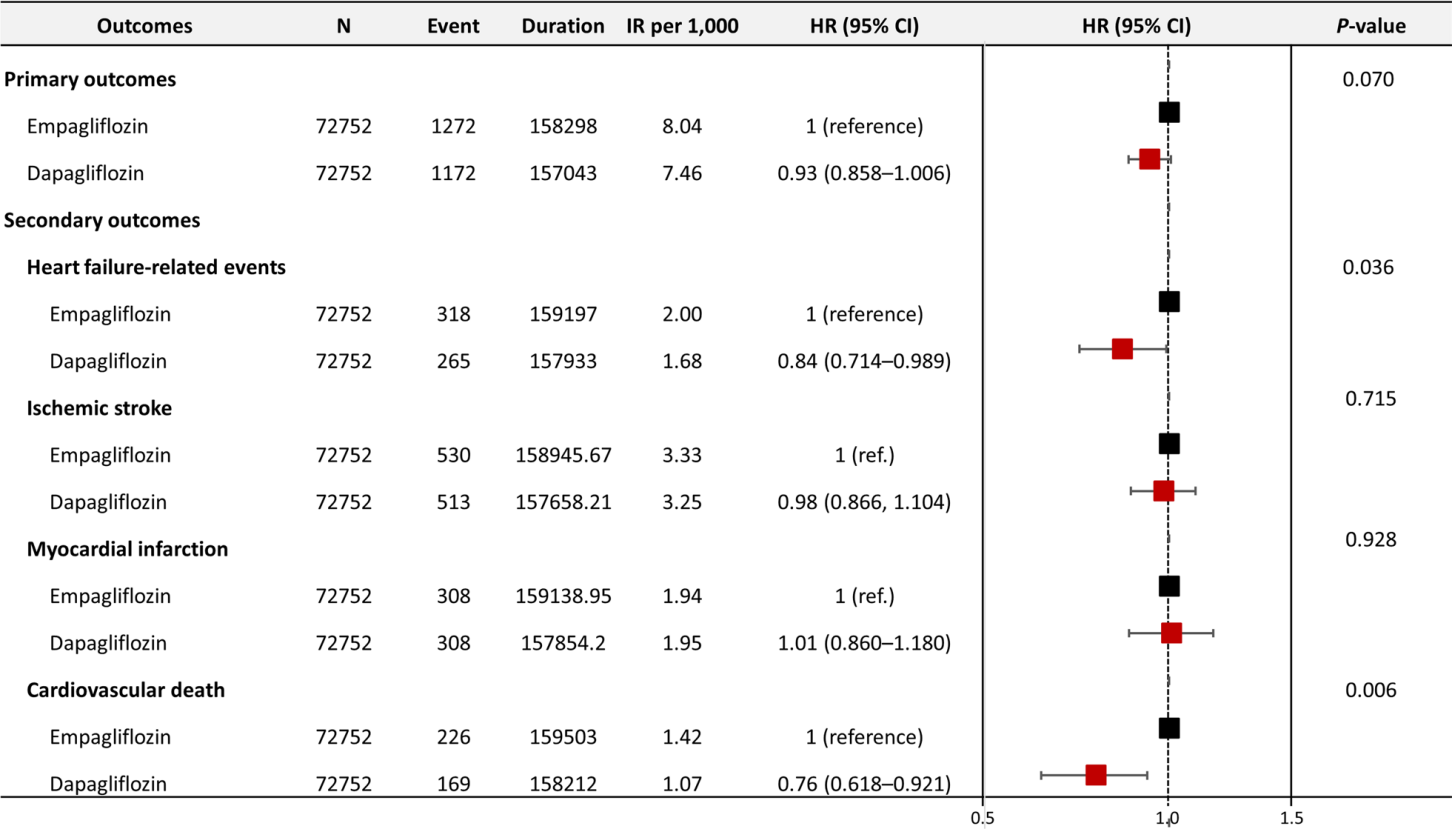
Forest plot of primary and secondary outcomes . The dashed vertical line indicates no difference between the dapagliflozin and empagliflozin users . * CI *confidence intervals, *HR *hazard ratio, *IR *incidence rate

**Fig. 3:**
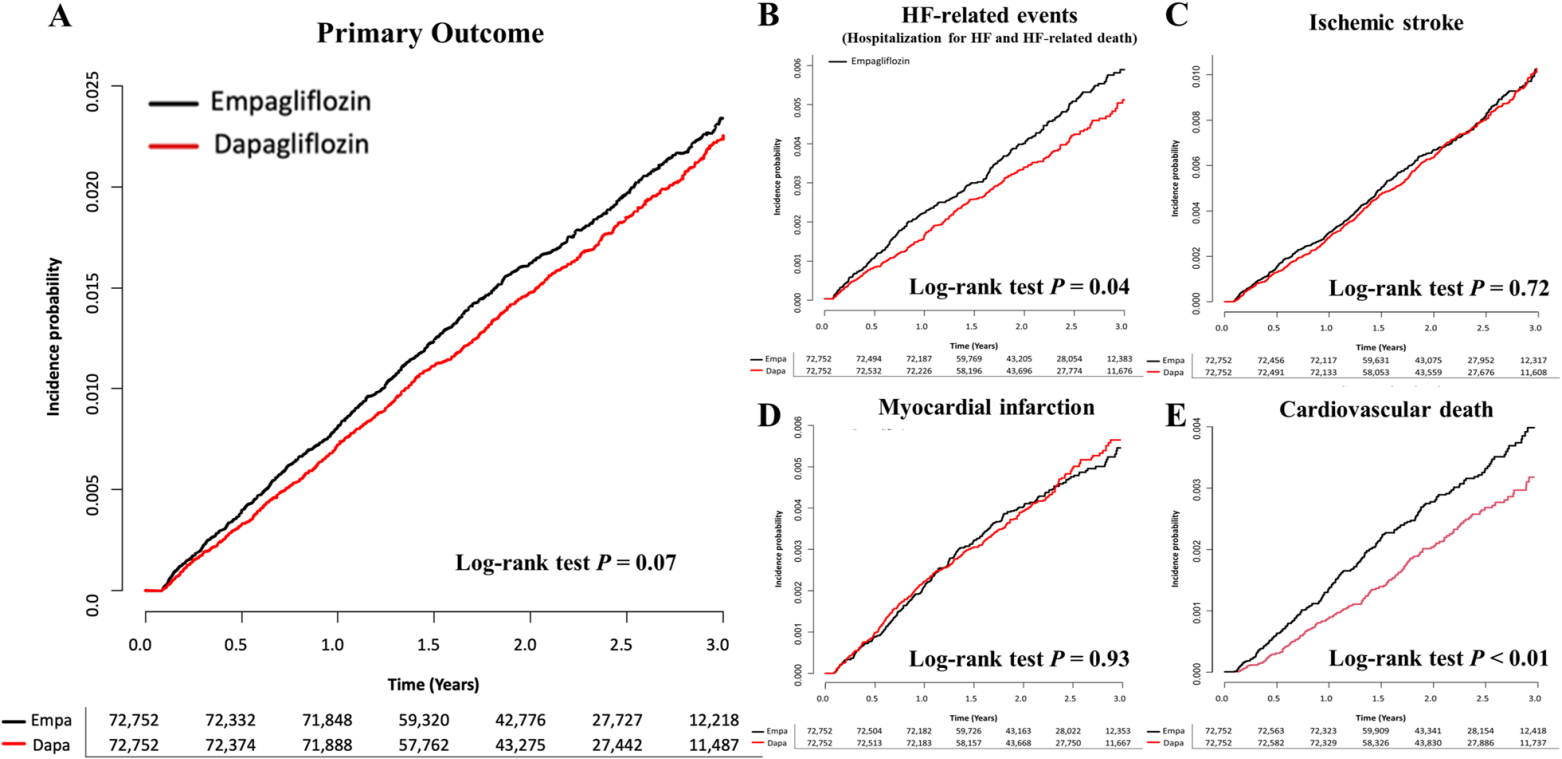
Kaplan–Meier curve of the incidence probability for the primary and secondary outcomes. No significant difference was observed in the incidence probability of the primary outcome between new dapagliflozin and empagliflozin users among patients with type 2 diabetes **A**. The primary outcomes included heart failure-related events, myocardial infarction, ischemic stroke, and cardiovascular death. Regarding the secondary outcomes, the risks of heart failure-related events **B**, ischemic stroke **C**, myocardial infarction **D**, and cardiovascular death **E** were compared

During a median follow-up of 2.08 (0.70) years, the primary outcome occurred in 2444 individuals, of which 1172 events were observed in dapagliflozin users and 1272 in empagliflozin users. The Kaplan–Meier curves of the incidence probability of the primary outcome did not significantly differ between both groups (HR 0.93, 95% CI 0.855–1.006, *P* = .07 by log-rank test, Figs. 2 ,  3 A).

### Secondary outcomes

The risks of heart failure-related events (HR 0.84, 95% CI 0.714–0.989, *P* = .036) and cardiovascular death (HR 0.76, 95% CI 0.618–0.921, *P* = .006) were significantly lower in dapagliflozin users than in empagliflozin users. In contrast, both groups did not significantly differ regarding ischemic stroke or MI (Figs. 2 ,  3B, C, D, E).

### Safety Outcomes

The risks of developing end-stage renal disease, diabetic ketoacidosis, hypoglycemia, and genitourinary tract infection during the follow-up were not significantly different (Additional file 1: Table S3).

### Subgroup and sensitivity analyses

In the subgroup analyses based on age, insulin use, chronic kidney disease, heart failure, duration of diabetes, and cardiovascular risks, the results followed the same overall trend, and no significant interactions were observed (all *P-*values for interaction > 0.05, Additional file 1: Table S4). Meanwhile, in the subgroup analysis by sex, female dapagliflozin users had a significantly lower risk of the primary outcome than female empagliflozin users (HR 0.85, 95% CI 0.738–0.968, *P-*for-interaction = 0.044). In contrast, the risk of the primary outcome did not differ between both groups in males.

The results were similar in an analysis that censored those who skipped SGLT2 inhibitors for > 28 days (as-treated approach, Additional file 1: Table S5). Again, non-cardiovascular mortality was similar between both groups (HR 1.08, 95% CI 0.988–1.190, *P* = .089).

## Discussion

In this large-scale nationwide cohort study, we compared the cardiovascular benefits of dapagliflozin and empagliflozin in a real-world setting. Over 70,000 respective dapagliflozin and empagliflozin users were included, with a median follow-up of > 150,000 PY. To the best of our knowledge, this is the largest population-based cohort study comparing the efficacy of different SGLT2 inhibitors. We demonstrated several key findings. (1) The risk of composite cardiovascular outcomes of heart failure-related events, ischemic stroke, MI, and cardiovascular death was similar. However, female dapagliflozin users appeared to benefit more. (2) Dapagliflozin use was associated with significantly lower risks of heart failure-related events and cardiovascular death, whereas the risks of ischemic stroke and MI were similar. (3) The risks regarding the safety outcomes were similar in both groups. These results were consistent in the main intention-to-treat analysis and the as-treated sensitivity analyses.

### Comparison with previous studies and interpretation

Previous meta-analyses encompassing large CVOTs have demonstrated that both dapagliflozin and empagliflozin have favorable composite cardiovascular outcomes of cardiovascular death, MI, and ischemic stroke, as well as hospitalization for heart failure over placebo in patients with T2DM [16, 17, 34, 35]. No significant difference was observed across the medication classes, whereas some heterogeneity was presented regarding individual outcomes. As highlighted by Darren et al., however, the patient characteristics of these trials differed notably; thus, comparing dapagliflozin and empagliflozin based on these trials might be limited [2–4, 16]. In particular, when comparing the DECLARE-TIMI 58 (Dapagliflozin Effect on Cardiovascular Events–Thrombolysis in Myocardial Infarction 58) trial to the EMPA-REG OUTCOME (Empagliflozin Cardiovascular Outcome Event Trial in Type 2 Diabetes Mellitus Patients–Removing Excess Glucose) trial, it should be noted that the latter included patients with much more comorbidities [2, 4]: even between the placebo groups of both studies, composite cardiovascular outcomes and cardiovascular death were two-fold and three-fold higher, respectively, in the EMPA-REG OUTCOME trial. In addition, since previous meta-analyses aggregated study-level data, they might be less appropriate to compare two medications at an individual level [16, 17, 34, 35]. In contrast, in Korea, both medications are approved for use in the same medical condition and are covered by medical insurance, resulting in a negligible difference in the actual cost paid by patients (< $10 per year). Consequently, which medication to prescribe is entirely at the individual physician’s discretion. More so, the baseline characteristics of both groups could have been similar even prior to PS matching. Therefore, this study might be advantageous when comparing the efficacies of both drugs.

Compared to previous meta-analyses that suggested no discernible difference among different SGLT2 inhibitors, this study showed that dapagliflozin might have similar but slightly favorable composite cardiovascular outcomes compared to empagliflozin and significantly lower risks of cardiovascular death and heart failure-related events [16, 17, 34, 35]. These findings might be attributed to significant differences in the study population. Large CVOTS encompassed in meta-analyses comprised mostly white males but fewer females and Asian ethnic groups. For instance, Asians comprised approximately 13.5% and 21.5% in the DECLARE-TIMI 58 and EMPA-REG OUTCOME, respectively. The mean age in both trials was 63–64 years, women comprised about 37% and 28%, and the mean BMI was 30.6 and 32.0 kg/m^2^, respectively. Conversely, the entire study population in our cohort was Asian. Women comprised 42%, and the mean BMI was 26.9 kg/m^2^. Most importantly, our study included a general T2DM population, and nearly two-fifths had low cardiovascular risks, whereas the aforementioned CVOTs included a highly selective population of T2DM patients with multiple cardiovascular risk factors or established cardiovascular diseases. Specifically, in the EMPA-REG OUTCOME trial, ≥ 75% of the study participants had evidence of ischemic heart disease, and nearly 25% had a documented history of stroke [2]. As a result, our study’s incidence rate for the composite of cardiovascular death, MI, and ischemic stroke was 6.03–6.44/1,000 PY—far lower than the 22.6/1000 PY of DECLARE-TIMI 58 or 37.4/1000 PY of EMPA-REG OUTCOME—demonstrating the significant difference in the study population. Accordingly, these differences might explain our novel findings. More importantly, given its substantial sample size associated with real-world data, our study can more accurately reflect actual clinical practice.

Meanwhile, a retrospective cohort study conducted in Taiwan showed consistent results with our findings with 12,681 new users of dapagliflozin or empagliflozin, i.e., similar risks of composite cardiovascular outcomes and a significantly lower risk of heart failure in dapagliflozin users [36]. Cardiovascular death in this Taiwanese study was also lower in the dapagliflozin group (HR 0.54, 95% CI 0.14–2.12). However, the difference was not significant, probably due to few events considering the wide CI. With a larger sample size, our study might corroborate the trend of a lower risk of cardiovascular death in the dapagliflozin group observed in the Taiwanese study. These consistent results are hypothesis-generating that dapagliflozin might have a drug-specific effect on Asians. Future studies are needed to identify associations between ethnic, cultural, or lifestyle differences and the pharmacokinetics and pharmacodynamics of dapagliflozin and empagliflozin.

As the SGLT2 inhibitor arm was superior to the placebo in terms of heart failure hospitalization and cardiovascular death in landmark clinical trials, it is intriguing to note that significant differences between users of dapagliflozin and empagliflozin were observed only for these two outcomes [2–4, 12–17]. This may allude to intrinsic disparities between the two drugs and could lead us to posit that dapagliflozin may exert more pronounced pleiotropic effects on heart failure outcomes than empagliflozin. For example, different neurohormonal responses might be one of the possible mechanisms. A previous study revealed that empagliflozin significantly increased plasma aldosterone and noradrenaline levels; however, dapagliflozin did not [37]. It was also noted that the change in plasma volume is a key mediator in reducing cardiovascular death among users of SGLT2 inhibitors [38]. Taken together, different neurohormonal responses and the concomitant reductions in plasma volume might synergistically contribute to the further decrease in the risk of heart failure-related events and cardiovascular death in dapagliflozin users. On the other hand, neurohormonal responses may not affect ischemic endpoints such as MI or ischemic stroke, as SGLT2 inhibitors have been found to be independent of pathways governing arterial thrombosis [39]. This is further supported by the pronounced advantages observed in women. Specifically, in our subsequent analysis, the cardiovascular benefits were evident only in women > 50 years. Although individual menopausal status could not be noted, women > 50 years, who were mostly in their peri- or post-menopausal stage and, hence, more susceptible to changes in neurohormones and plasma volume, would have benefited more from dapagliflozin and showed improved cardiovascular outcomes [40]. Secondly, the approximate 2.5-fold greater affinity for SGLT2 and six-fold greater affinity for SGLT1 exhibited by dapagliflozin compared to empagliflozin might have contributed [41]. Currently, the benefits of adding SGLT1 to SGLT2 inhibition are receiving attention. A study with Mendelian randomization data revealed that patients with missense mutations in the SGLT1 gene had reduced risks of heart failure and death [42]. Recent clinical trials, SOLOIST (effect of sotagliflozin on cardiovascular events in patients with type 2 diabetes post worsening heart failure) and SCORED (effect of sotagliflozin on cardiovascular and renal events in patients with type 2 diabetes and moderate renal impairment who are at cardiovascular risk), also raised the possible advantage of additional SGLT1 inhibition for cardiovascular outcomes [43, 44]. Accordingly, the higher SGLT1 and SGLT2 affinity of dapagliflozin compared to that of empagliflozin might contribute to the lower risks of heart failure-related events and cardiovascular death in our study. In addition, several studies have suggested that the SGLT2-independent effects of this drug class, which are presumed to take place in the myocardium, are likely attributed to off-target effects, given the notably low levels of SGLT2 in cardiac cells [45]. Further investigations focusing on differences in off-target effects between dapagliflozin and empagliflozin are needed.

### Limitations

Our study had several limitations. First, there might have been potential residual confounders that were not taken care of. For example, we lacked data regarding medications that might affect the study outcome, such as aspirin or angiotensin receptor-neprilysin inhibitors. Second, this study mainly included Koreans. Thus, generalizing the study results to other racial/ethnic groups may be challenging. Third, our study did not include dosage data. Nevertheless, considering that the HRs for cardiovascular outcomes were similar for 10 mg and 25 mg of empagliflozin in the EMPA-REG OUTCOME trial, missing dosage data are unlikely to change our results significantly [2]. Finally, adherence to the treatment regimen could not be confirmed. However, similar results in the intention-to-treat and as-treated sensitivity analyses and the large sample size in this study would mitigate the potential bias.

## Conclusion

Our study presents the cardiovascular outcomes of SGLT2 inhibitors—dapagliflozin and empagliflozin—in Asian patients with T2DM. The risk of composite cardiovascular outcomes did not differ between new dapagliflozin and empagliflozin users. However, the risks of heart failure-related events and cardiovascular death might be lower in dapagliflozin users.

## Supplementary information

**Additional file 1: Table S1.** Definition of outcomes and covariate. **Table S2.** Baseline characteristics of study population before PS matching. **Table S3.** Safetyoutcomes. **Table S4.** Subgroup analysis. **Table S5.** Sensitivity analysis.      

## Data Availability

Raw data, sample cohort data, or customized cohort data are all available from designated terminals if approved by the NHIS. Those unauthorized are restricted from accessing the data.
