# Supplementary material for: Comparative cardiovascular outcomes in type 2 diabetes patients taking dapagliflozin versus empagliflozin: a nationwide population-based cohort study
Source: Cardiovasc Diabetol. 2023 Jul 26;22:188. doi: 10.1186/s12933-023-01911-7 (PMC10373410; doi:10.1186/s12933-023-01911-7)
Supplement: Supplementary file 1 — Additional file 1: Table S1. Definition of outcomes and covariate. Table S2. Baseline characteristics of study population before PS matching. Table S3. Safetyoutcomes. Table S4. Subgroup analysis. Table S5. Sensitivity analysis. [file 12933_2023_1911_MOESM1_ESM.docx]

**Table S1. Definition of outcomes and covariates**

**Table S2. Baseline characteristics of study population before PS matching**

**Table S3. Safety outcomes**

**Table S4. Subgroup analysis**

**Table S5. Sensitivity analysis**

**Table S1. Definition of outcomes and covariates**

| **Covariates** | **Descriptions** |
| --- | --- |
| **Study population** |  |
| Type 2 diabetes | Admission ≥ 1 or outpatient clinic ≥ 2 for E11–E14 as a primary diagnosis  with  Minimum 1 prescription of anti-diabetic drugs (sulfonylureas, metformin, meglitinides, thiazolidinediones, dipeptidyl peptidase-4 inhibitors, α-glucosidase inhibitors, SGLT2-inhibitor, GLP-1 agonist, or insulin) |
| **Study outcomes** |  |
| Heart failure-related events |  |
| Hospitalization for heart failure | Admission ≥ 1 for I50, I42.0, I11.0, I13.0, I13.2 as primary diagnosis |
| Death from heart failure | Cause of death from I50, I42.0, I11.0, I13.0, I13.2 |
| Ischemic stroke | Admission ≥ 1 for I63, I64 as primary diagnosis with claims for brain CT or MRI  with Procedure codes: HA441, HA451, HA461, HA851 for brain CT; HE101, HE201, HE301, HE401, HE501 for brain MRI |
| Myocardial infarction | Admission ≥ 1 for I21 or I22 as primary diagnosis  with  coronary artery bypass graft or procedure code (HA670) of coronary angiography |
| Cardiovascular death | Cause of death I000-I999 |
| **Safety outcomes** |  |
| End-stage renal disease | 1) Have procedure code for ≥90 days after the first occurrence of that procedure code: O7011-7020 for hemodialysis, O7071-O7075 for peritoneal dialysis  OR  2) Special code of V001 for hemodialysis, V003 for peritoneal dialysis, or V005 for kidney transplantation  OR  3) Procedure code R3280 (Kidney transplantation) |
| Diabetes ketoacidosis | E10.1, E11.1, E12.1, E13.1 |
| Genitourinary tract infection | N10, N15.1, N30, N34, N37.0, N39.0 for urinary tract infection, and B37.3, B37.4, N77.1, N76 for genital infection among women |
| Hypoglycemia | E 16.x, E1163, E1363, and E1463 |
| **Covariates** |  |
| Hypertension | Admission ≥ 1 or outpatient clinic ≥ 2 for I10-I13, I15 as a primary diagnosis Minimum 1 prescription of anti-hypertensive drug (thiazide, loop diuretics, aldosterone antagonist, alpha-/beta-blocker, calcium-channel blocker, angiotensin-converting enzyme inhibitor, angiotensin II receptor blocker) |
| Dyslipidemia | Admission ≥ 1 or outpatient clinic ≥ 2 for E78 as a primary diagnosis  Minimum 1 prescription of lipid-lowering medication (statin, ezetimibe, fenofibrate) |
| Chronic kidney disease | Estimated glomerular filtration rate <60 ml/min/1.73m^2^ |
| Heart failure | Admission ≥ 1 or outpatient clinic ≥ 2 for I50, I42.0, I11.0, I13.0, and I13.2 as a primary diagnosis |
| Peripheral artery disease | Admission ≥ 1 or outpatient clinic ≥ 2 for I70.2, I73 as a primary diagnosis |
| Atrial fibrillation | Admission ≥ 1 or outpatient clinic ≥ 2 for I48 as a primary diagnosis |
| Chronic obstructive pulmonary disease | Admission ≥ 1 or outpatient clinic ≥ 2 for J43-44 as a primary diagnosis |
| Liver cirrhosis | Admission ≥ 1 or outpatient clinic ≥ 2 for K70.3, K74.3-74.6, K71.1, K76.1 |
| Hyperthyroidism | Admission ≥ 1 or outpatient clinic ≥ 2 for E05 as the primary diagnosis |

Abbreviation: CT, computed tomography; GLP-1, glucagon-like peptide-1; MRI, magnetic resonance imaging; SGLT2, sodium-glucose co-transporter-2.

**Table S2. Baseline characteristics of study population before PS matching**

| **Variables** | **Empagliflozin** | **Dapagliflozin** | **ASD** |
| --- | --- | --- | --- |
|  | N=73,110 | N=118,064 |  |
| **Age, years** | 56.2 ± 11.1 | 55.1 ± 11.0 | 0.099 |
| **Sex, male** | 42523 (58.2) | 67971 (57.6) | 0.012 |
| **Index year** |  |  |  |
| 2016 | 12664 (17.32) | 40953 (34.69) | 0.404 |
| 2017 | 31360 (42.89) | 41629 (35.26) | 0.157 |
| 2018 | 29086 (39.78) | 35482 (30.05) | 0.205 |
| **Systolic blood pressure, mmHg** | 128.4 ± 14.9 | 128.2 ± 14.8 | 0.012 |
| **Diastolic blood pressure, mmHg** | 78.6 ± 10.1 | 78.8 ± 10.0 | 0.016 |
| **Body mass index, kg/m^2^** | 26.9 ± 4.0 | 27.0 ± 4.0 | 0.043 |
| **Duration of diabetes, years** | 7.1 ± 5.5 | 6.8 ± 5.4 | 0.068 |
| **Low Income** | 14805 (20.3) | 23607 (20.0) | 0.006 |
| **Urban residents** | 31324 (42.9) | 50085 (42.4) | 0.009 |
| **Smoking status** |  |  |  |
| Never smoker | 39226 (53.7) | 63760 (54.0) | 0.007 |
| Ex-smoker | 16185 (22.1) | 25611 (21.7) | 0.011 |
| Current smoker | 17699 (24.2) | 28693 (24.3) | 0.002 |
| **Alcohol drinking status** |  |  |  |
| Never drinker | 42855 (58.6) | 68583 (58.1) | 0.011 |
| Mild drinker (< 30g/day) | 24155 (33.0) | 39242 (33.2) | 0.004 |
| Heavy drinker (≥ 30g/day) | 6100 (8.3) | 10239 (8.7) | 0.012 |
| **Regular exercise** | 15283 (20.9) | 23966 (20.3) | 0.015 |
| **Comorbidities** |  |  |  |
| Hypertension | 42738 (58.5) | 66880 (56.7) | 0.037 |
| Dyslipidemia | 53584 (73.3) | 84305 (71.4) | 0.042 |
| Heart failure | 3797 (5.2) | 5098 (4.3) | 0.041 |
| Myocardial infarction | 2113 (2.9) | 2534 (2.2) | 0.047 |
| Peripheral artery disease | 16397 (22.4) | 25274 (21.4) | 0.025 |
| Ischemic stroke | 2106 (2.9) | 3096 (2.6) | 0.016 |
| Atrial fibrillation | 1976 (2.7) | 2364 (2.0) | 0.046 |
| COPD | 7114 (9.7) | 11060 (9.4) | 0.012 |
| Liver cirrhosis | 610 (0.8) | 1069 (0.9) | 0.009 |
| Hyperthyroidism | 2186 (3.0) | 3525 (3.0) | 0.000 |
| **Medication** |  |  |  |
| ARB/ACE inhibitor | 38630 (52.8) | 60626 (51.4) | 0.011 |
| Beta-blocker | 8038 (11.0) | 11140 (9.4) | 0.051 |
| Calcium channel blocker | 23578 (32.3) | 36757 (31.1) | 0.024 |
| Diuretics | 10180 (13.9) | 15898 (13.5) | 0.013 |
| **GLD before use of SGLT2 inhibitor** |  |  |  |
| Metformin | 68430 (93.6) | 110429 (93.5) | 0.003 |
| Sulfonylurea | 39637 (54.2) | 63569 (53.8) | 0.008 |
| Meglitinides | 359 (0.5) | 648 (0.55) | 0.008 |
| Thiazolidinedione | 10950 (15.0) | 17646 (15.0) | 0.001 |
| DPP4 inhibitor | 46053 (63.0) | 72432 (61.4) | 0.034 |
| α-glucosidase inhibitor | 1557 (2.1) | 2873 (2.4) | 0.020 |
| Insulin | 10715 (14.7) | 15577 (13.2) | 0.043 |
| GLP-1 agonist | 632 (0.9) | 784 (0.7) | 0.023 |
| Numbers of GLD ≥3 | 34637 (47.4) | 54737 (46.4) | 0.020 |
| **GLD in combination with SGLT2 inhibitor** |  |  |  |
| Metformin | 61804 (84.5) | 98814 (83.7) | 0.023 |
| Sulfonylurea | 28579 (39.1) | 45666 (38.7) | 0.008 |
| Meglitinides | 28 (0.04) | 65 (0.06) | 0.009 |
| Thiazolidinedione | 1263 (1.7) | 1584 (1.3) | 0.032 |
| DPP4 inhibitor | 5397 (7.4) | 10328 (8.8) | 0.050 |
| α-glucosidase inhibitor | 121 (0.2) | 256 (0.2) | 0.011 |
| Insulin | 5638 (7.7) | 9268 (7.9) | 0.005 |
| GLP-1 agonist | 51 (0.07) | 85 (0.07) | 0.000 |
| Numbers of GLD ≥ 3 | 30489 (41.7) | 47587 (40.3) | 0.028 |
| **Estimated glomerular filtration rate** | 92.5 ± 48.2 | 93.7 ± 50.9 | 0.024 |
| <60 ml/min/1.73m^2^ | 5413 (7.4) | 7431 (6.3) | 0.044 |
| 60–90 ml/min/1.73m^2^ | 32477 (44.4) | 52112 (44.1) | 0.006 |
| ≥90 ml/min/1.73m^2^ | 35220 (48.2) | 58521 (49.6) | 0.028 |
| **Urine protein by dipstick test** |  |  |  |
| Negative | 62555 (85.6) | 101447 (85.9) | 0.011 |
| Trace | 3719 (5.1) | 6170 (5.2) | 0.006 |
| Positive | 6836 (9.4) | 10447 (8.9) | 0.017 |
| **Serum laboratory test** |  |  |  |
| Fasting plasma glucose, mg/dL | 157.0 ± 55.2 | 159.4 ± 57.1 | 0.042 |
| Total cholesterol, mg/dL | 181.9 ± 46.0 | 184.3 ± 46.3 | 0.053 |
| Hemoglobin, mg/dL | 14.4 ± 1.6 | 14.4 ± 1.63 | 0.024 |

Data are presented in number (percentage) for categorical variables and mean ± standard deviation for continuous variables.

Abbreviation: ACE, angiotensin-converting enzyme; ASD, absolute standardized difference; ARB, Angiotensin II receptor blocker; COPD, chronic obstructive pulmonary disease; DPP4, dipeptidyl peptidase-4; GLD, glucose-lowering drug; GLP-1, glucagon-like peptide-1; PS, propensity score; SGLT2, sodium-glucose co-transporter-2.

**Table S3. Safety outcomes**

| **Outcomes** | **Event** | **IR per 1,000** | **HR (95% CI)** | ***P*-value** |
| --- | --- | --- | --- | --- |
| **End-stage renal disease** |  |  |  |  |
| Empagliflozin | 106 | 0.66 | 1 (reference) | 0.544 |
| Dapagliflozin | 96 | 0.61 | 0.92 (0.696–1.210) |  |
| **Diabetic ketoacidosis** |  |  |  |  |
| Empagliflozin | 113 | 0.71 | 1 (reference) | 0.982 |
| Dapagliflozin | 112 | 0.71 | 1.00 (0.768–1.295) |  |
| **Genitourinary tract infection** |  |  |  |  |
| Empagliflozin | 14106 | 101.94 | 1 (reference) | 0.534 |
| Dapagliflozin | 14110 | 103.12 | 1.01 (0.984–1.031) |  |
| **Hypoglycemia** |  |  |  |  |
| Empagliflozin | 618 | 3.89 | 1 (reference) | 0.712 |
| Dapagliflozin | 626 | 3.98 | 1.02 (0.914–1.141) |  |

Abbreviation: CI, confidence interval; HR, hazard ratio; IR, incidence rate.

**Table S4. Subgroup analysis**

| **Subgroups** | **SGLT2 inhibitor** | **Number of subjects** | **Event** | **IR per 1,000** | **Adjusted HR (95% CI)** | ***P***  **for interaction** |
| --- | --- | --- | --- | --- | --- | --- |
| **Age** |  |  |  |  |  |  |
| <65 years | Empagliflozin | 57060 | 639 | 5.13 | 1 (reference) | 0.560 |
|  | Dapagliflozin | 57894 | 622 | 4.99 | 0.97 (0.866–1.081) |  |
| ≥65 years | Empagliflozin | 15692 | 633 | 18.73 | 1 (reference) |  |
|  | Dapagliflozin | 14858 | 550 | 16.92 | 0.92 (0.823–1.035) |  |
| **Sex** |  |  |  |  |  |  |
| Male | Empagliflozin | 42300 | 810 | 8.80 | 1 (reference) | 0.044 |
|  | Dapagliflozin | 42403 | 785 | 8.74 | 1.00 (0.910–1.108) |  |
| Female | Empagliflozin | 30452 | 462 | 6.87 | 1 (reference) |  |
|  | Dapagliflozin | 30349 | 387 | 5.76 | 0.85 (0.738–0.968) |  |
| **Insulin use** |  |  |  |  |  |  |
| No | Empagliflozin | 67132 | 1073 | 7.32 | 1 (reference) | 0.497 |
|  | Dapagliflozin | 66906 | 971 | 6.72 | 0.94 (0.857–1.020) |  |
| Yes | Empagliflozin | 5620 | 199 | 16.89 | 1 (reference) |  |
|  | Dapagliflozin | 5846 | 201 | 16.02 | 1.01 (0.827–1.226) |  |
| **Chronic kidney disease** |  |  |  |  |  |  |
| No | Empagliflozin | 67394 | 1003 | 6.83 | 1 (reference) | 0.409 |
|  | Dapagliflozin | 67880 | 961 | 6.56 | 0.97 (0.883–1.054) |  |
| Yes | Empagliflozin | 5358 | 269 | 23.45 | 1 (reference) |  |
|  | Dapagliflozin | 4872 | 211 | 19.81 | 0.89 (0.739–1.062) |  |
| **Duration of diabetes** |  |  |  |  |  |  |
| <7 years | Empagliflozin | 37587 | 394 | 4.83 | 1 (reference) | 0.099 |
|  | Dapagliflozin | 38292 | 387 | 4.76 | 1.01 (0.877–1.161) |  |
| ≥7 years | Empagliflozin | 35165 | 878 | 11.44 | 1 (reference) |  |
| (Continued) |  |  |  |  |  |  |
| **Subgroups** | **SGLT2 inhibitor** | **Number of subjects** | **Event** | **IR per 1,000** | **Adjusted HR (95% CI)** | ***P***  **for interaction** |
|  | Dapagliflozin | 34460 | 785 | 10.36 | 0.92 (0.832–1.010) |  |
| **History of heart failure** |  |  |  |  |  |  |
| No | Empagliflozin | 69029 | 1000 | 6.64 | 1 (ref.) | 0.072 |
|  | Dapagliflozin | 69213 | 956 | 6.40 | 0.96 (0.882-1.053) |  |
| Yes | Empagliflozin | 3723 | 272 | 35.26 | 1 (ref.) |  |
|  | Dapagliflozin | 3539 | 216 | 28.28 | 0.80 (0.671-0.960) |  |
| **Cardiovascular risk group** |  |  |  |  |  |  |
| Low cardiovascular risk | Empagliflozin | 27977 | 147 | 2.40 | 1 (ref.) | 0.994 |
|  | Dapagliflozin | 29355 | 140 | 2.24 | 0.93 (0.741-1.177) |  |
| Multiple cardiovascular risks | Empagliflozin | 14686 | 244 | 7.56 | 1 (ref.) |  |
|  | Dapagliflozin | 14603 | 229 | 7.17 | 0.95 (0.793-1.137) |  |
| Established cardiovascular diseases | Empagliflozin | 30089 | 881 | 13.6 | 1 (ref.) |  |
|  | Dapagliflozin | 28794 | 803 | 12.8 | 0.94 (0.857-1.038) |  |

Abbreviation: CI, confidence interval; HR, hazard ratio; IR, incidence rate; SGLT2, sodium-glucose co-transporter-2.

**Table S5. Sensitivity analysis**

| **Outcomes** | **28 days censoring** | | | | | |
| --- | --- | --- | --- | --- | --- | --- |
|  | **N** | **Event** | | **IR per 1,000** | **HR (95% CI)** | ***P*-value** |
| **Primary outcome** |  |  | |  |  |  |
| Empagliflozin | 72752 | 717 | | 6.82 | 1 (reference) |  |
| Dapagliflozin | 72752 | 659 | | 6.47 | 0.95 (0.854–1.055) | 0.333 |
| **Secondary outcomes** |  |  |  |  |  |  |
| **Heart failure-related events** |  |  |  |  |  |  |
| Empagliflozin | 72752 | 165 | | 1.56 | 1 (reference) |  |
| Dapagliflozin | 72752 | 127 | | 1.24 | 0.79 (0.630–1.000) | 0.050 |
| **Ischemic stroke** |  |  |  |  |  |  |
| Empagliflozin | 72752 | 306 | | 2.90 | 1 (ref.) |  |
| Dapagliflozin | 72752 | 293 | | 2.87 | 0.991 (0.844, 1.163) | 0.908 |
| **Myocardial infarction** |  |  |  |  |  |  |
| Empagliflozin | 72752 | 175 | | 1.66 | 1 (ref.) |  |
| Dapagliflozin | 72752 | 185 | | 1.81 | 1.09 (0.885–1.339) | 0.420 |
| **Cardiovascular death** |  |  |  |  |  |  |
| Empagliflozin | 72752 | 125 | | 1.18 | 1 (reference) |  |
| Dapagliflozin | 72752 | 92 | | 0.90 | 0.76 (0.581–0.995) | 0.046 |

Abbreviation: CI, confidence interval; HR, hazard ratio; IR, incidence rate.
